# Pineal cysts without hydrocephalus: microsurgical resection via an infratentorial-supracerebellar approach—surgical strategies, complications, and their avoidance

**DOI:** 10.1007/s10143-022-01831-2

**Published:** 2022-07-12

**Authors:** Steffen Fleck, Ahmed El Damaty, Ina Lange, Marc Matthes, Ehab El Rafaee, Sascha Marx, Jörg Baldauf, Henry W. S. Schroeder

**Affiliations:** 1 Department of Neurosurgery, University Medicine Greifswald, Ferdinand-Sauerbruch-Straße 1, 17475 Greifswald, Germany; 2 Department of Neurosurgery, University Heidelberg, Heidelberg, Germany; 3 Department of Neurosurgery, Kasr Alainy Faculty of Medicine, Cairo University, Cairo, Egypt; 4 Department of Cancer Immunology and Virology, Dana-Farber Cancer Institute, Harvard Medical School, Boston, MA USA

**Keywords:** Pineal cyst, Neurosurgery, Microsurgery, Complication, Supracerebellar-infratentorial approach

## Abstract

Indications for surgery of pineal cysts without ventriculomegaly are still under debate. In view of the limited data for pineal cyst resection in the absence of hydrocephalus, and the potential risk of this approach, we have analyzed our patient cohort focusing on strategies to avoid complications according to our experience in a series of 73 pineal cyst patients. From 2003 to 2015, we reviewed our database retrospectively for all patients operated on a pineal cyst. Furthermore, we prospectively collected patients from 2016 to 2020. In summary, 73 patients with a pineal cyst were treated surgically between 2003 and 2020. All patients were operated on via a microscopic supracerebellar-infratentorial (SCIT) approach. The mean follow-up period was 26.6 months (range: 6–139 months). Seventy-three patients underwent surgery for a pineal cyst. An absence of enlarged ventricles was documented in 62 patients (51 female, 11 male, mean age 28.1 (range 4–59) years). Main presenting symptoms included headache, visual disturbances, dizziness/vertigo, nausea/emesis, and sleep disturbances. Complete cyst resection was achieved in 59/62 patients. Fifty-five of 62 (89%) patients improved after surgery with good or even excellent results according to the Chicago Chiari Outcome Scale, with complete or partial resolution of the leading symptoms. Pineal cysts resection might be an indication in certain patients for surgery even in the absence of ventriculomegaly. The high percentage of postoperative resolution of quality-of-life impairing symptoms in our series seems to justify surgery. Preoperatively, other causes of the leading symptoms have to be excluded.

## Introduction

Magnetic resonance imaging (MRI) reveals pineal cysts in 1.8 to 4.3% of healthy subjects [1–4]. Autopsy studies suggest that up to 40% of the general population harbors a pineal cyst [5–7]. Although usually asymptomatic, pineal cysts can grow leading to mass effect on the surrounding structures (tectum, aqueduct, venous structures) and thereby necessitate treatment [2, 8–19].

Due to the high prevalence of pineal cysts with nonspecific symptoms, the discussion about the relation of the symptoms to the pineal cysts is ongoing [6, 20–23]. Nowadays, the management of patients who present only with headache but without neurological symptoms and a pineal cyst in the absence of hydrocephalus is not clearly defined. Some authors suggest that in the absence of obstructive hydrocephalus or tectal compression, symptoms should not be attributed to the pineal cyst and these patients should be managed conservatively [20]. Otherwise, it is postulated that pineal cyst may cause intermittent obstruction of the Sylvian aqueduct or compression of critical neural structures, making resection reasonable after thorough preoperative examination [2, 6, 12, 24–29]. Pineal abnormalities may play a critical role in sleep disturbances and even in major depressive disorders [30]. Due to ethical reasons, a randomized controlled trial will be not acceptable to clarify more insights in the role of surgical therapy for pineal cysts associated with “unspecific symptoms.” Large prospective studies are limited, whereas prospective data collection of case series seems to be a proper tool [31].

Therefore, we present our experience with the resection of pineal cysts in the absence of ventriculomegaly to roll out the indication for surgery and to report on complications and their avoidance.

## Patients and methods

The study protocol has been approved by the local ethics committee. It includes a retrospective review of prospectively collected data and records of the patients who underwent a surgery for a pineal cyst without ventriculomegaly from 2003 to 2015 as well as a follow-up clinical examination. All subjects gave written informed consent. Furthermore, we collected data prospectively from 2016 to 2020. We studied our consecutive case series from a single academic institution regarding demographic data, presenting symptoms, radiological characteristics, surgical approach, extent of resection, perioperative complications, as well as clinical and radiological follow-up. Other possible and obvious causes of the leading symptoms had been ruled out by consultations (e.g., ophthalmology, neurology, neuropediatrics, internal medicine, psychiatry, and/or psychology). The investigations were carried out in the referring center. Otherwise, we completed the missing investigations in our institution within a structured preoperative work-up. Intracranial pressure (ICP) monitoring was not performed prior to surgery.

In view of the still vague indication for surgery in pineal cysts without hydrocephalus, we discussed extensively the decision with our patients individually. Typical symptoms considered during decision-making were headache associated with symptoms denoting elevated intracranial pressure, i.e., nausea and/or emesis, visual disturbance (blurring of vision, upward gaze palsy) resulting from possible tectal compression. Other, more unrelated symptoms were sometimes described by patients (see Table 1 for details).

**Table 1:** Characteristics of patients, type and position of surgery

Signs/symptoms	*n*	%
Headache	61	98.4%
Nausea	39	62.9%
Dizziness/vertigo	25	40.3%
Sleeping disorder	17	27.4%
Blurred vision	16	25.8%
Diplopia	10	16.1%
Emesis	8	12.9%
Malaise	6	9.7%
Concentration deficits	6	9.7%
Ataxia	4	6.4%
Tinnitus	2	3.2%
Memory deficits	2	3.2%
Vomiting	1	1.6%
Upward gaze paresis	1	1.6%
Syncope	1	1.6%
Generalized hypesthesia	1	1.6%
Facial hemihypesthesia	1	1.6%
Dysphagia	1	1.6%
Attack of sweating	1	1.6%
Age	Mean 28.1 yrs (± 12.1)	Min 4 to max 59
Sex	Female	Male
	51 (82.3%)	11 (17.7%)
Type of surgery	MSR	MTR
	4 (6.4%)	58 (93.6%)
Position of surgery	CC	SS
	10 (16.1%)	52 (83.9%)

*MTR* microsurgical total resection, *MSR* microsurgical subtotal resection, *SS* semi-sitting position, *CC* Concorde position

Importantly, the difficulty in attributing (all) their symptoms to the pineal cyst, and that their symptoms or at least part of them may have resulted from a temporary elevation in intracranial pressure was extensively discussed with the patients. Especially, that they may not benefit from surgery, and on the contrary, being exposed to the risks of surgery.

### Surgery

Preoperatively, transesophageal echocardiography was performed to rule out an open foramen ovale and to minimize the risks from air embolism. The surgical notes were reviewed regarding the approach, characteristics of cyst removal, and perioperative complications as mentioned above.

### Postoperative evaluation

All patients from 2016 to 2020 were assessed prospectively after surgery and before discharge, 3 and 6 months after surgery and then yearly. All patients were followed finally until April 2021. The examinations were carried out by a neurosurgeon who was not directly involved in the surgery. The Chicago Chiari Outcome Scale [32] (CCOS, see Table 2) was calculated.

**Table 2:** The Chicago Chiari Outcome Scale (CCOS) [32]: a total score of 4–16; a final score of 4: incapacitated; 8: impaired outcome; 12: functional outcome; 16: excellent outcome. A cut-off score of 11 to denote a better or worse outcome [32]

Chicago Chiari Outcome Scale				
Pain	Non-pain	Functionality	Complications	Total score
1: Worse	1: Worse	1: Unable to attend	1: Persistent complication, poorly controlled	4: Incapacitated outcome
2: Unchanged and refractory to medication	2: Unchanged or improved but impaired	2: Moderate impairment (< 50% attendance)	2: Persistent complication, well controlled	8: Impaired outcome
3: Improved or controlled with medication	3: Improved but unimpaired	3: Mild impairment (> 50% of attendance)	3: Transient complication	12: Functional outcome
4: Resolved	4: Resolved	4: Fully functional	4: Uncomplicated course	16: Excellent outcome

Májovsky et al. used this score to assess the clinical outcome of patients with symptomatic pineal cysts as they have some similarities with patients with Chiari malformation [33].

### Radiological evaluation

The diameter of the pineal cyst was measured as maximum width of the cyst in a mid-sagittal T2-weighed or constructive interference in steady state (CISS) MR image.

### Surgical details

#### Approach and positioning

**Fig. 1:**
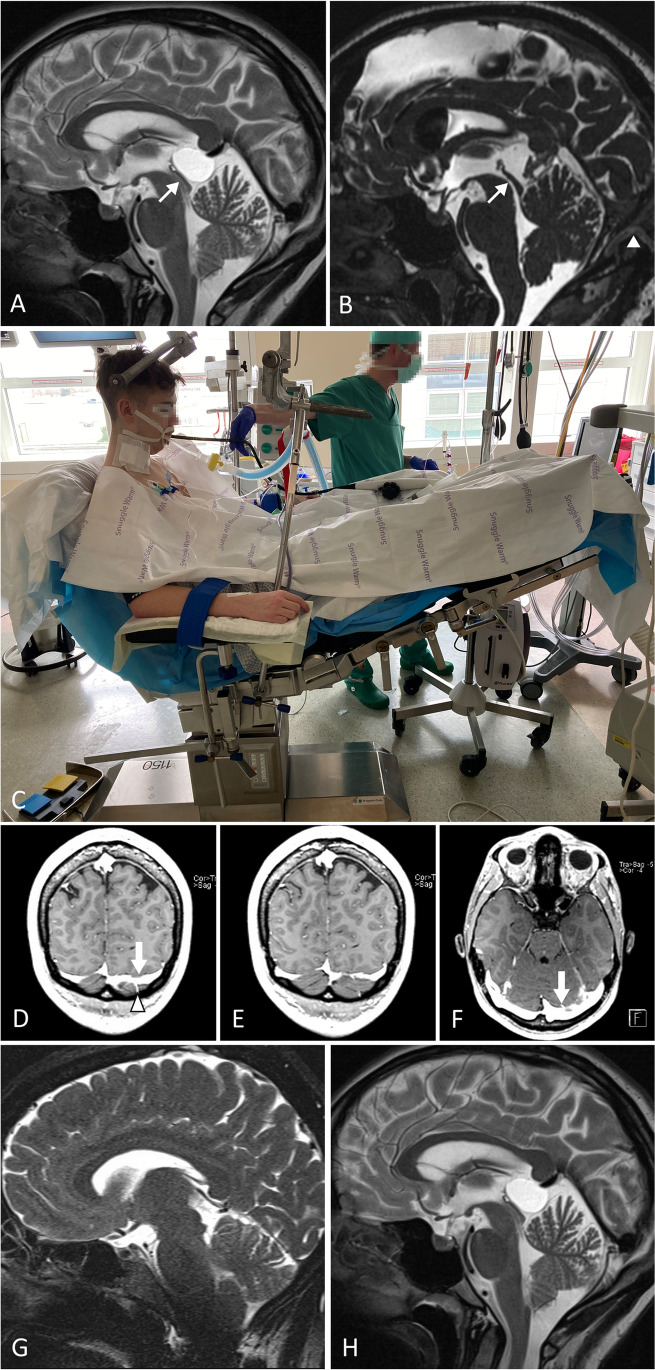
Midsagittal MR CISS before (**A**) and after surgery (**B**) demonstrating a pineal cyst leading to a narrowed Sylvian aqueduct (arrow) without hydrocephalus. Patient complaining of longstanding and increasing headache attacks. Post-operative MR CISS (**B**) midsagittal image: complete cyst resection; open Sylvian aqueduct. Craniotomy reaching the transverse sinus (arrow-head). Symptoms resolved completely. **C** Semi-sitting position. Contrast-enhanced T1-weighted MR (coronal (**D**, **E**), axial (**F**)): left-sided predominant sinus and bridging vein; decision for approach on right side with the higher-rising sinus. Variations of steepness of tent: paramedian (**G**) viewing trajectory to the pineal region is not as steep as in the midline (**H**) approach

A microsurgical total cyst resection was performed via a supracerebellar-infratentorial approach (SCIT). Initially, we used the standard midline approach (*n* = 15). However, since 2015 we have used the less invasive small unilateral paramedian approach. This approach has the advantage that the viewing trajectory to the pineal region is not as steep as in the midline approach (see Fig. 1G).

In 53 individuals, a semi-sitting positioning has been performed. Regarding a persistent foramen ovale (in preoperative transesophageal echocardiography), surgery was done in prone position (*n* = 9) (Fig. 1C).

#### Specific anesthesiological considerations

Specific anesthesiological preparations include central venous line and permanent transesophageal echocardiographic monitoring to offer the direct control of an ongoing air embolism.

#### Preoperative planning and radiographic landmarks

We use anatomical landmarks to perform the approach. Navigation was not applied.

We look carefully for any side predominance and height of the transverse sinus as well as the location of bridging veins. In children, we look additionally for an open occipital sinus. If one transverse sinus is high rising at the coronal MR image, we approach from that side because the angle of view is not so steep.

MR imaging with contrast-enhanced T1-weighted and T2-weighted images in axial and coronal plane shows the sinus and veins very well (see Fig. 1D–F). The steepness of the tent is very variable (see Fig. 1G, H).

We prefer a paramedian 2-cm wide supracerebellar approach on the non-dominant side of a high-rising transverse sinus, ideally with smaller or absent bridging veins.

#### Skin incision, bone flap

**Fig. 2:**
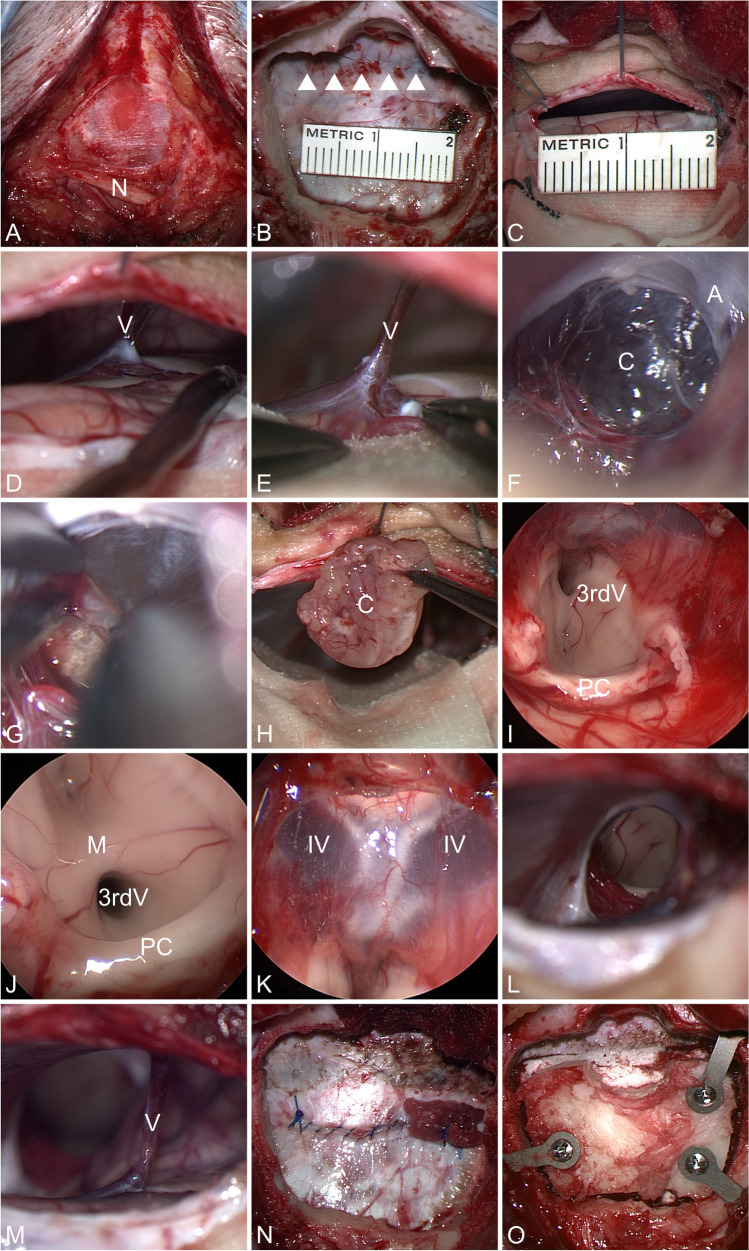
**A** Skin incision; greater occipital nerve (N). **B** Craniotomy exposing the transverse sinus. **C** Dural incision alongside the transverse sinus. **D** and **E** Dissection of bridging vein (V). **F** Pineal cyst (C) surrounded by thick arachnoid (A). **G** Bimanual dissection. **H** Resected pineal cyst (C). **I** + **J** Endoscopic view into the third ventricle showing massa intermedia (M) and posterior commissure (PC). **K** Endoscopic view to the roof of the third ventricle with a 45° endoscope shows the large internal cerebral veins (IV). **L** Microscopic view of the resection cavity showing gross total cyst resection. **M** Preservation of the bridging vein after cyst removal. **N** Dural closure. **O** Bone flap fixation with miniplates

A straight 5–6 cm long skin incision beginning 1.5 cm over the level of the external occipital protuberance 2 cm paramedian to the midline is made. The fascia is incised and with careful sharp and blunt dissection we look for the major occipital nerve (Fig. 2A). Then, a 2–3 cm paramedian bone flap exposing the lower third of the transverse sinus is raised (Fig. 2B). After the craniotomy, a jugular compression test is performed to make sure that no venous leak exists.

#### Opening of the dura, CSF drainage, and bridging veins

The dura is incised below and parallel to the transverse sinus (Fig. 2C). Dural tacking stitches are placed to elevate the upper dural edge. This allows a view along the tent without any active retraction because of cerebellar retraction by gravity. Spatulas are not required. Careful covering of bone and dural edges after meticulous hemostasis is mandatory. If somehow possible, we avoid occluding even smaller veins. If a larger vein is within the approach trajectory to the pineal gland, we dissect the vein from the cerebellar surface to allow a little bit of cerebellar retraction by gravity to enable access to the pineal cyst while avoiding vein sacrifice (Fig. 2D, E, F).

The arachnoid covering the pineal region is always thick. Before arachnoid incision, the large veins such as vein of Galen and Rosenthal need to be identified. If they are collapsed, jugular compression should be performed. After opening of the quadrigeminal cistern, CSF is drained. Surgeon’s patience while draining enough CSF is a key step for a gentle operation.

#### Dissection of the cyst

The internal cerebral and basal veins and many of their tributaries converge at the great cerebral vein (of Galen) in the posterior incisural space. The vein of the cerebellomesencephalic fissure crosses the quadrigeminal cistern (ambient cistern) and drains directly or via superior vermian vein to the vein of Galen. Arterial tributaries are defined as P3 segment of PCA (posterior cerebral artery) and also enter the quadrigeminal cistern [34]. After opening of the quadrigeminal cistern (ambient cistern), the cyst is exposed by meticulous sharp dissection (Fig. 2G). Always, a good arachnoid dissection plane can be found around the cyst. The cyst is incised and collapsed. Typically, the cysts contain a yellowish fluid. The cyst wall is grasped with a small tumor forceps and the arachnoid plane is dissected with an anatomical forceps (traction-counter traction technique) [35]. Small feeding and draining vessels are coagulated and cut. The cyst is dissected from superior and inferior colliculi (quadrigeminal plate) beneath, from the thalamus laterally, and from habenula and posterior commissure anteriorly. After cyst removal (Fig. 2H), a jugular compression test is done to detect potential venous bleeding sources.

Endoscopic visualization (Fig. 2I–K) incl. 30° and 45° optics is of help to look to surrounding structures. However, in view of the usually narrow surgical corridor and the intended preservation of bridging veins, we do not use a purely endoscopic technique since the risk of damaging the veins is much higher because they are not in the visual field of the endoscope.

#### Closure

The dura is closed with a running suture (Fig. 2N). Usually, we add sealants (e.g., Hemopatch®, Baxter; Tachosil®, Takeda) to prevent any cerebrospinal fluid (CSF) leak via the small stitch channels. Rarely, we use a small muscle patch in larger dural defects. Interestingly, despite the opening of the third ventricle, we rarely observed (see Table 3) CSF leaks after surgery. The bone flap is fixed with titanium miniplates (Fig. 2O). The incision is closed in layers.

**Table 3:** Detailed complications

Complication	*n*	Therapy (*n*)/remarks
Pneumothorax	2	Thoracal drainage (1)
Air embolism (intraoperative)	8	Relevant with circulatory impairment (2); venous aspiration
Pneumocephalus	Nearly all	Spontaneous resolution
Hydrocephalus	0	
Discrete circumscribed abnormalities on diffusion MRI postop	5	Cerebellar (5) without clinical sequelae
Discrete media infarction (due to slight air embolism??)	1	(Transient) slight foot paresis
Rebleeding	0	
Meningitis	0	
Local wound infection	1	Antibiotics
CSF fistula	2	Subcutaneous effusion; spontaneous resolution
Occipital hypesthesia	1	Only numbness
Diplopia/visual disturbances	11	Spontaneous resolution
New mnestic deficits	2	
Ataxia	0	
Increased sleep disturbance	2	Improved with melatonin
Tinnitus	1	Spontaneous resolution
Peripheral nerve	3	Numbness (occipital nerve) (2), weakness (sciatic nerve) (1) spontaneous resolution

## Results

### Patients and presenting symptoms

Around 500 patients with pineal cysts were referred to our institution for evaluation from 2003 to 2020. Seventy-three patients underwent surgery for a pineal cyst. An absence of enlarged ventricles was documented in 62 patients (51 female, 11 male, mean age 28.1 years (± 12.15 years), range 4–59 years).

Presenting symptoms included headache (61/62), visual disturbances (23/62), dizziness/vertigo (24/62), nausea/emesis (41/62), and sleep disturbances (17/62). All the neuro-ophthalmologic examinations could rule out any objective preoperative disturbances.

### Radiological findings

Preoperative mid-sagittal MRI (CISS or T2-weighted) usually demonstrated narrowing but no occlusion of the aqueduct due to the pineal cyst (Fig. 1A). The cyst size ranged from 7 to 27 mm (mean 14.87 mm) in maximal diameter. Enlarged ventricles were not observed. There was radiological evidence of cyst enlargement in preoperative follow-up MRI series in 6 of 62 patients. MRI obtained 1 day after surgery demonstrated no cyst remnant in all patients. Furthermore, there was no cyst recurrence during the follow-up period (mean 26.6 months (range: 6–139 months)).

### Clinical outcome

Fifty-five of 62 (89%) patients improved after surgery with good or even excellent results according to the Chicago Chiari Outcome Scale (CCOS ≥ 12), with complete or partial resolution of the leading symptoms within a mean follow-up period of 26.6 months (range: 6–139 month). Details of outcome are shown in Table 1.

The average hospital stay was 7 days (± 3.5 days, range 5–22 days). The longer hospital stays were due to completion of the preoperative investigations. All patients were discharged to their homes. Neither direct referrals to rehabilitation centers nor long-term care facilities were needed.

### Surgery

The semi-sitting position was used in 53 patients. Otherwise, we operated them in prone position in case of persistent oval foramen (*n* = 9).

In all patients, a microsurgical cyst resection was performed via a supracerebellar-infratentorial approach (SCIT). Total cyst resection could be achieved in 59 of 62 patients. Initially, we used the standard midline approach (*n* = 15). However, since 2015 we have used a less invasive small unilateral paramedian approach (*n* = 47).

The operation time was in average 211 (± 51) min (range 108 to 346 min).

### Peri- and postoperative complications

Air embolism occurred in 8 patients. In only 2 of these patients, we encountered a significant air embolism with circulatory impairment during surgery which was managed with jugular vein compression to visualize the venous leak, and aspiration of air from the heart via the central line. In the other 6 patients, air embolism was only detected by the cardiac ultrasound. In all patients, air embolism had no sequelae. Intraoperative major surgical complications were not observed.

Pathology approved all resected cysts as simple pineal cysts without evidence of malignancy.

Eleven patients suffered from transient visual problems after surgery (diplopia in 5 patients, blurring of vision in 4 patients, nystagmus (*n* = 1), and 1 visual field defect). However, a detailed neuro-ophthalmological examination after surgery did not demonstrate any deficit in these patients. These symptoms resolved completely within 2–4 weeks after surgery in all patients.

One patient suffered from a wound infection and was managed conservatively.

Two subcutaneous CSF effusions occurred and resolved spontaneously. A postoperative hydrocephalus was not observed.

Postoperatively, analgetic medications have been used stepwise according to the WHO guidelines. In 2 patients, who had problems to fall into sleep, melatonin was administered for 2 to 4 weeks. No patient received long-term melatonin medication.

Further detailed information is shown in Table 3.

## Discussion

To our knowledge, this series is the largest surgical series of patients who underwent resection of a simple pineal cyst without hydrocephalus. Most of our patients showed a good to excellent outcome which is in accordance with the literature.

Evaluating CCOS, in our series 55 of 62 (89%) patients reported good outcome with more than 11 points. In all patients, ventriculomegaly was not present suggesting that the absence of ventriculomegaly and/or tectum induced visual disturbances (Parinaud’s syndrome) is not a contraindications for surgery. Therefore, a surgical intervention should not be generally refused in case of an absent ventriculomegaly in patients with leading symptoms indicating a possible temporary increase of intracranial pressure.

Pineal cysts rarely cause symptoms [20, 36, 37]. They are usually assumed as incidental findings and were often identified during workup for headache. Mostly, they are not the cause of symptoms [20, 21, 38, 39]. According to the literature, large pineal cysts are considered to be potentially symptomatic lesions [6] while causing ventriculomegaly due to compression of the Sylvian aqueduct or Parinaud’s syndrome due to tectal compression [6, 10, 12, 15, 24, 25, 28, 29, 40–42].

Therefore, the discussion about the best management of patients with a pineal cyst without clear signs and symptoms of intracranial hypertension or symptomatic mass effect is still ongoing. There are some case series describing surgical results in pineal cysts without hydrocephalus or tectal compression. Kalani et al. [6] reported in 17 of 18 patients with resection of pineal cysts a resolution or improvement of their presenting symptoms. The authors suggest that the absence of ventriculomegaly and Parinaud’s syndrome are not absolute contraindications to surgical intervention.

Our previous results (series of 43 patients operated on pineal cyst without enlarged ventricles) could already demonstrate good to excellent clinical results [43]. In this period (2003 to 2018), we examined and followed 440 patients with pineal region pathologies according to Diagnosis Related Groups (DRG) codes.

However, we should clearly state that most pineal cysts are asymptomatic and do not require treatment. It is illustrated by Al-Holou and coworkers [20] who reviewed in one of the largest studies more than 48,000 adult patients undergoing brain MRI: they discovered 478 incidental pineal cysts greater than 5 mm on MRI. Headache was presented in 21% of them, but no cysts were considered as symptomatic, and surgical intervention was not indicated. During follow-up, 99% of cysts remained stable or even decreased in size.

Obviously, patients should be thoroughly investigated before deciding for a surgical resection in case of such a benign lesion and in view of the potential risk in this approach. Nevertheless, we believe that our results indicate that surgery may be an option after careful selection of patients to offer the possibility of marked relief of their symptoms and better quality of life in a small subset of pineal cyst patients.

Similar results were presented by Eide and Ringstad [44] who collected 27 patients managed surgically for non-hydrocephalic pineal cysts over a 10-year period.

The compression of the internal cerebral veins, vein of Rosenthal, or vein of Galen may influence the severity of symptoms in subjects with pineal cysts [45]. The resulting central venous hypertension might cause mild edema within the territories drained by the affected veins. MRI biomarkers indicating central venous hypertension have recently been reported [46].

Májovsky et al. reported over 110 patients with simple pineal cysts. The most common presenting symptoms were tension headache (62.7%), vertigo (16.4%), migraine (12.7%), syncope (10.9%), nausea (8.2%), and diplopia (8.2%). Twenty-one patients underwent pineal cyst resection; 20 patients (95.2%) reported some improvement in their presenting symptoms, and 10 patients (47.6%) were completely free after the surgery [33]. Their results of the surgical group are comparable to our results after surgery. We reported in 95% a good outcome after surgery and a very low incidence of complications [43]. Constantly, we can also demonstrate a low rate of complications with our recent cohort.

Recently, Masina et al. published a meta-analysis of the published literature addressing the surgical treatment of symptomatic pineal cysts without hydrocephalus. They concluded that although the results support the role of surgery in the management of this entity, they have to be interpreted with a great deal of caution as the current evidence is limited, consisting only of case reports and retrospective surgical series. Inherent to such studies are inhomogeneity and incompleteness of data, selection bias, and bias related to assessment of outcome carried out by the treating surgeon in the majority of cases. Prospective studies with patient-reported and objective outcome assessment are needed to provide higher level of evidence [47].

Some aspects of the surgical approach have to be discussed.

The SCIT represents an extraparenchymatous approach via a natural corridor. Alternatives might be the occipital transtentorial approach (OTT) and the occipital bi-transtentorial/falcine approach [34] or—only in case of enlarged ventricles—the frontal endoscopic approach. We would not suggest the OTT because the pineal cyst is located under the tent and veins. Why should we come from above with the need to cut the tent? Furthermore, there is a risk of postoperative hemianopia [48–50]. Also, pure endoscopic SCIT has been described [2]. However, the narrow surgical corridor in our minimally invasive paramedian approach does not allow an unrestricted manual dissection with an endoscope.

We prefer the (semi-) sitting position (Fig. 2): advantages are proper gravity-induced cerebellar retraction and fluid drainage which avoids a limited view in the depth and the need for suction. Otherwise, the prone position offers a safe alternative in view of the risk of air embolism.

To leave small amounts of pineal tissue behind in view of preservation of melatonin production has been suggested previously [33], but we usually perform a total cyst removal. We have only seen a few temporary sleep problems which were treated with a temporary melatonin substitution. Furthermore, it is not proven that a small piece of cyst will work physiologically.

### Key issues on selection of patients

We assumed that the leading symptoms (headache, nausea, vomiting) of the patients are caused by a temporary increase of the intracranial pressure due to the partial aqueduct compression. During attacks of emesis, the intracranial pressure might be markedly elevated so that the compression of the aqueduct is overcome. All other potential causes for the complaints must be excluded. All patients had been thoroughly investigated prior to surgery in view of the leading symptoms (ophthalmology, neurology or neuropediatrics, internal medicine, neuropsychology, psychiatry) to rule out any other possible cause for their symptoms. We have to clearly state that the decision for resection of a simple pineal cyst without ventriculomegaly has to be taken with caution and after a long discussion with the patient and failure of all other conservative measures to control his or her symptoms.

### Limitation

We could not rule out any placebo effect. It was pointed out that young female patients are more susceptible to placebo effect [51]. Most of our series were young females. However, the long-term success in most of our patients as well as the improvement in our pediatric cohort makes a placebo effect very unlikely. The patient cohort is still a subject to selection bias as most of the patients with pineal cysts and without symptoms do not seek medical attention. Furthermore, we have to state that our study is a case study (level of evidence 4 according to the Oxford Centre of Evidence-based Medicine).

## Conclusion

To date, scientific information about surgical therapy of pineal cysts without hydrocephalus has been limited. We suggest that pineal cysts resection can be done also in the absence of ventriculomegaly in selected patients. The high percentage of postoperative resolution of quality of life limiting symptoms in our series demonstrates a good indication for pineal cyst resection. Preoperatively, other causes of the leading symptoms have to be excluded. Intermittent occlusion of the aqueduct may cause increased intracranial pressure leading to intermittent symptoms. Furthermore, a possible venous hypertension due to compression of the deep venous structures through the pineal cyst may also be responsible for the symptoms. However, the decision-making remains difficult since no reliable predictors are available. There is still insufficient scientific evidence to generally recommend this technique as a treatment for headache and other unspecific symptoms.

## Data Availability

There is availability of data and material on demand.

## Code availability

Software application or custom code: not applicable.

## Abbreviations

CCOS: Chicago Chiari Outcome Scale
CISS: Constructive Interference in Steady State
CSF: cerebrospinal fluid
DRG: Diagnosis Related Groups
MSR: microsurgical subtotal resection
MTR: microsurgical total resection
PEEP: positive endexspiratory pressure
SCIT: supracebellar infratentorial approach
SS: semi-sitting position
CC: concord position
